# Local Attitudes towards Bear Management after Illegal Feeding and Problem Bear Activity

**DOI:** 10.3390/ani3030935

**Published:** 2013-09-12

**Authors:** Sara Dubois, David Fraser

**Affiliations:** Animal Welfare Program, University of British Columbia, 2357 Main Mall, Vancouver, BC, V6T 1Z4, Canada; E-Mail: david.fraser@ubc.ca

**Keywords:** attitudes, black bear, British Columbia, Christina Lake, food-conditioned, human-wildlife conflict, intentional feeding, telephone survey

## Abstract

**Simple Summary:**

The “pot bears” received international media attention in 2010 after police discovered the intentional feeding of black bears during the investigation of an alleged marijuana-growing operation in Christina Lake, British Columbia. Residents of this small community were surveyed by phone twice over the following year, before and after government actions. This study aimed to understand local attitudes on how these bears should be managed and whether they differed from existing bear management policy. Results indicate a significant problem with the public view of wildlife feeding and a gap between public and expert opinion on relocation and killing of food-conditioned wildlife.

**Abstract:**

The “pot bears” received international media attention in 2010 after police discovered the intentional feeding of over 20 black bears during the investigation of an alleged marijuana-growing operation in Christina Lake, British Columbia, Canada. A two-phase random digit dialing survey of the community was conducted in 2011 to understand local perspectives on bear policy and management, before and after a summer of problem bear activity and government interventions. Of the 159 households surveyed in February 2011, most had neutral or positive attitudes towards bears in general, and supported the initial decision to feed the food-conditioned bears until the autumn hibernation. In contrast to wildlife experts however, most participants supported relocating the problem bears, or allowing them to remain in the area, ahead of killing; in part this arose from notions of fairness despite the acknowledged problems of relocation. Most locals were aware of the years of feeding but did not report it, evidently failing to see it as a serious form of harm, even after many bears had been killed. This underscores the importance of preventive action on wildlife feeding and the need to narrow the gap between public and expert opinion on the likely effects of relocation *versus* killing.

## 1. Introduction

Many lessons have been learned over decades of bear conflict management in North America [1,2,3], with the response of government agencies to human-bear interactions evolving over time [4]. Although expert-driven policy has generally directed bear management in British Columbia [5], the public has increasingly come to expect participatory decision-making in both resource and wildlife management, facilitated by the growth of social science research in the fields [6,7,8]. A potential benefit of increasing public participation is that an understanding of public attitudes may lead to better communication of management goals and greater support for the actions taken [9,10]. 

In August 2010, the story of more than 20 Canadian “pot bears” appeared in the international media after a police raid of a property near Christina Lake, a seasonal tourist town in the interior of British Columbia (BC) near the United States border, with fewer than 1,000 permanent residents. The investigation discovered an alleged marijuana-growing operation and numerous docile black bears, seemingly curious about the presence of the police [11]. Photographs and reports of police standing beside the bears quickly dominated local news and spread to international news agencies [12,13,14]. Media stories spoke of a “Bear Lady” living in the woods [15] and an online video showed “The Bear Dude” [16] who described feeding dog food to the bears for years, and denied reports that the bears were being used to “guard” the marijuana production.

Upon hearing that generations of bears had been fed on the property for over two decades, members of the public (mostly non-local) began creating online petitions and Facebook pages to “Save the Christina Lake Bears.” Public concern that the bears would be killed by the authorities was legitimate as there are limited non-lethal options for dealing with highly *habituated* bears that are *food-conditioned* (definitions as per [2]). Further, the current provincial *problem* bear policy supports killing such bears rather than relocating them [17]. Finally, black bears do not hold any special conservation status in BC; the estimated population of 120,000–160,000 province-wide is considered healthy, and several thousand are hunted annually [18]. In fact, BC has one of the highest black bear populations in North America; the government receives about 10,000 bear complaints annually [4], and several hundred problem black bears are killed annually as a result of human-bear conflict [19]. Therefore, killing these particular food-conditioned bears was a likely option for wildlife officials. 

However, contrary to current policy and laws against feeding dangerous wildlife, the government agency responsible for bear management in BC decided to allow the food-conditioned bears to continue to be fed by the resident until they started hibernation, given the time of year (late summer), the complexity of the issue (high number of bears, criminal court case pending), and significant media attention [20]. However, the authorities required that more natural foods be introduced to the diet (such as fruits and vegetables) and that all feeding cease after hibernation. Although this was an unusual case for wildlife managers in BC, human encroachment into wildlife habitat and a wide availability of attractants has meant that some black bears populations are becoming increasingly reliant on anthropogenic food sources [21], creating more potential for human-bear conflict. Most problem black bear incidents result from the unintentional feeding of bears that access fruit trees, garbage and other attractants [3,21,22], rather than intentional feeding as in this case.

There were many media reports on opinions of the broader public about saving the food-conditioned bears, but little was known about the views of Christina Lake locals. Therefore, a random digit dialing telephone survey of the community was initiated to understand participant opinions and perceptions of local bears. As attitudes can change over time and with experience, the survey was repeated after the initial management intervention (lethal removal of problem bears). As management decisions directly affect local residents, who may or may not share the views of the greater public and/or experts, the telephone study was able to take an active approach by surveying local attitudes on the highly publicized problem bear issue as it was still unfolding. 

The first survey aimed to understand attitudes and beliefs about bears in general; tolerance levels specifically towards the food-conditioned bears before they awoke from hibernation (*i.e.*, before any possible interaction between the community and the bears); and levels of public support for wildlife management options and penalties for intentional feeding. The second survey with the same individuals was designed to see whether attitudes towards the food-conditioned bears, management options, and penalties changed after a spring, summer and fall during which there would be a high potential for bear activity and human-bear conflict. 

## 2. Methods

### 2.1. Survey Design

Given the quantity of data desired, the limited time for pre- and post-intervention questioning, and uncertainty of ensuring a sufficient number of responses to less personal surveys (mail or internet), a telephone survey was selected as the research tool. Further, mail surveys can be costly and time consuming [23] and an internet survey was not chosen due to difficulty in targeting only Christina Lake residents and the unknown extent of internet usage in this remote area. However, a telephone directory of households was publicly available for random sampling. Cost effective and easy to administer [24], telephone surveys face considerable challenges in an era of growing technology [24,25,26,27]. Despite these concerns, this method was a valid option for this study as the remote mountainous location has limited mobile telephone service and most homes continue to have a land line.

In the first (conducted in February 2011, 8 multi-part questions) and second survey (conducted in December 2011, 8 multi-part questions), participants were asked to describe their general bear sighting experiences as either “positive” (uneventful, comfortable), “negative” (uncomfortable, stressful), or “neutral”. A five-point scale (*1 = not supportive at all* to *5 = very supportive*) was used to measure support for varying management strategies for the food-conditioned bears. Both closed and open questions were asked and bears were referred to in the survey calls as either “general bears” or the “fed bears”. Answers to closed questions were designed to be analyzed in a pre-coded format (e.g., male = 0, female = 1) [28]. Calls were not recorded, but qualitative responses and post-survey comments were noted verbatim and some are presented as quotes.

The first question in the initial survey asked about length of residency in Christina Lake to determine the respondent’s eligibility for the survey [29] and gender of respondent was noted. The next question asked if there was a registered hunter in the household. Questioning then progressed from questions about frequency of bear sightings and safety concerns in general, to questions on attitudes towards past and future management strategies specifically for the food-conditioned bears (listed in Table 2). Finally, an open-ended question on what respondents felt would be an appropriate penalty for feeding these bears was asked. 

The second survey was administered to the same person who participated in the first survey within the sampled household. It focused on observed bear activity within the past year, including any problem bear activity and attitudes towards this. Given that all participants knew the location of the bear feeding, they were asked how far in kilometers they lived from this property. Participants were also asked to score recent management strategies for the food-conditioned bears and advise what strategies they would recommend to other communities facing similar circumstances. Also respondents were asked if they agreed or disagreed with the proposed legal charges for feeding the bears (as full penalty details were not known at this time). As part of survey development, the questions were reviewed with a government biologist familiar with the bear issue and a polling company manager with expertise in public opinion surveys. 

### 2.2. Sampling and Recruitment

As individuals within one household likely would have discussed this well-known local event, household rather than individual was selected as the research unit. A household was categorized as a “hunting household” if it included at least one registered hunter, whether or not that individual was still active. The 2006 Christina Lake census reported 475 households in the area, averaging 2 individuals per household [30] and only adult (over 18 years of age) permanent inhabitants were surveyed. To achieve a sample size with 5–10% margin of error and 95% confidence level, the random digit dialing survey would require a sample between 81 and 213 households [29].

In order to randomize potential participants, residential telephone numbers listed in the 2010 print and 2011 online telephone directories for Christina Lake were entered into a spreadsheet and assigned a random number, which was then sorted from lowest to highest. Interviewers were given a sequential page of new numbers for each call session and/or a list of numbers from previous uncompleted call attempts. To advise participants about the first survey and to help legitimize participation, newspaper advertisements were published in the Christina Lake News (print) and The Boundary Sentinel (online) to inform the community that they might be contacted to participate in research survey by telephone. 

### 2.3. Data Collection and Analysis

Telephone numbers were called in the randomized order until the call was completed or five attempts were made [24,31]. Calling sessions totalled 100 hours for the first survey (calls to all area non-business phone numbers) and 60 hours for the second (only calls to past participants), and included a variety of weekdays, weekday evenings, and weekend sessions. Interviewers logged all call attempts. For completed calls, interviewers read the survey script verbatim, ensured eligibility (adult, Christina Lake resident), briefed respondents on their anonymity and confidentiality, and received verbal consent before recording answers. Although respondents were anonymous during the survey, they were given the option of providing a first name or nickname to be reached for the second survey. 

Quantitative results produced descriptives and frequencies compiled in SPSS, which was also used to calculate inferential statistics [32]. *T*-tests were used to assess differences by gender and hunting activity in attitudes towards management options as evaluated by the five-point scale. A paired *t*-test was used to determine any change in participants’ attitudes towards penalties for feeding. Chi-squared tests were used to assess residents’ attitudes towards bears and support for management actions based on the distance they lived from the property of concern. Qualitative analysis of text responses and comments involved inductive content analysis [33]. Reporting of comments was categorized as “few” (<5% of participants), “some” (<50%), “many” (50–75%) and “most” (>75%) participants. Quotes (reported in italics) were chosen as representative statements of important themes or unique noteworthy insights. 

## 3. Results

### 3.1. Recruitment

In the first survey, 1478 call attempts were made to 610 telephone numbers listed in the directories. Ineligible numbers included business numbers (33), non-working numbers (143), numbers with no eligible respondent (3), and hearing impairment or language barrier (2). Unknown number status (150) included all “no answer” and answering machines. Of the remaining 279 eligible and known working numbers, 159 households completed the survey (for +/− 6.13 margin of error) and 120 declined. The response rate of 43% for the first survey was calculated according to the method of CASRO [31]. To help determine non-response bias, respondent gender was noted and showed a similar ratio for refused surveys (45% male, 55% female) as for completed surveys (39% male, 61% female) (*p* > 0.05, Fisher’s Exact test). The gender ratio of refused surveys did not differ significantly from that of the 2006 census data of the area (50% male, 50% female) [30] (*p* > 0.05, Fisher’s Exact test).

In the second survey, a maximum of five attempts were also made to the same 159 households. Of these, there were 150 eligible and known working numbers with 123 households completing the second survey, 3 declining, and 24 were unreachable, for a CASRO response rate of 82% of original respondents.

### 3.2. Participants

Since results could reflect household attitudes on the issue and not just individual opinions, few individual demographic variables were collected. In the first survey, 61% of respondents were female, 75% of households were non-hunting, and 86% had lived in the area for more than 10 years. Demographic data were almost identical in the second survey, as 123 of the 159 original participants were re-surveyed. All respondents had heard of the bear feeding incident from media or other locals, and many said they knew it was going on long before the recent media attention. Several respondents recounted anecdotes about the well-known residents buying excessive amounts of dog food and about bears following these individuals into town. 

### 3.3. Awareness of Bears and Attitudes towards Bears in General

When asked in the first survey how frequently respondents observed general bear activity in 2010, signs of bears (scat, markings, tracks, damage) were seen more than once by 82% of respondents and actual bears on properties were observed more than once by 69%. Most respondents reported seeing bears in the overall Christina Lake area in the past year. Usually, respondents related bear sightings to the presence of garbage, compost or fruit trees on their properties. Many respondents indicated that local residents were more accepting of the bears’ presence than “*summer visitors*”. Some respondents said they had seen fewer bears in 2010 than previous years, while others suggested they had seen more. Respondents noted that the local landfill, where “*dump bears*” had often been seen and tolerated, was converted to a transfer station in 2010, likely leaving these bears without their usual source before winter.

When asked in the second survey about general bear activity over the past year, 50% of respondents stated they observed more bear activity in the general Christina Lake area in 2011 over 2010, but 32% saw about the same and only 18% saw less. Sightings of bears on respondents’ properties in 2011 were also reported as higher than in 2010 by 45% of respondents, while 39% saw about the same and only 16% saw less. Further, signs of bears (scat, markings, tracks, damage) on respondents’ properties were also reported to be seen more frequently in 2011 over the previous year by 44% of respondents, while 45% experienced about the same amount of signs and 11% observed less.

When asked to describe their bear sighting experience as positive, negative or neutral, most respondents in the first survey had a mixed, neutral-to-positive attitude with a “*live and let live*” approach towards bears and said it was normal for living near natural bear habitat (Table 1). Some respondents indicated they were more cautious as bears approached settled areas, while others had dogs and fences they felt gave them some protection. Although many respondents had generally positive views of local bears, some expressed negative attitudes towards the bears if they would come onto their properties. A few suggested they did not walk in areas known for high bear activity as they felt in general that bears were not afraid of people. Significant concerns over safety were expressed by a few that experienced bear destruction on their properties (e.g., destroying sheds, appliances) or attempts to enter a home.

Although attitudes towards both seeing bears and signs of bears on their property were negative, signs of bears were qualified as an “*annoyance*” related to having to clean up feces, being messy, or damage to trees and fences rather than fear of the bears’ presence. Many respondents shared concerns captured by this participant:

“Seeing a bear on your property is both positive and negative. Positive because they are wonderful animals and negative because of the damage they cause. They can be annoying because of the damage but they are nice to have around.”

In the second survey, fewer of the same respondents felt positive about seeing bear signs and bears, both on their property and in the area (Table 1). Most notably, more respondents felt negative towards seeing bears in Christina Lake and more felt neutral towards seeing bears on their property, in 2011 than in 2010 (*p* < 0.05 Fisher’s Exact test). Many respondents knew of serious damage caused to property that was not related to garbage, compost or fruit trees, including destruction of sheds, trailers, cabins and cars. Several unusual reports of very persistent bears seeking food, and bears opening doors and entering vehicles, were cited as examples of learned behaviour by the food-conditioned bears. Compared to respondents who lived at least 10 km from the property, those who lived within 5 km of the property were more likely to have generally negative attitudes towards (1) bears in the Christina Lake area, (2) bears on their property, and (3) signs of bears on their property, as determined by a Chi-squared test (*p* < 0.01) in each case.

**Table 1:** Percentage of respondents stating that they felt positive, neutral or negative towards general bear sightings across two surveys (February and December 2011) ^a^.

Attitudes	Bears in general Christina Lake area		Bears on own property		Signs of bears on own property	
Survey	First	Second	First	Second	First	Second
Positive	40% *	16% *	27% *	15% *	17%	13%
Neutral	43%	40%	33% *	43% *	42%	47%
Negative	17% *	44% *	40%	42%	41%	40%

^a^ Based on the 123 respondents who completed both surveys; * Significant difference between first and second surveys (*p* < 0.05) by Fisher’s Exact test.

### 3.4. Bear Activities in 2011

During the summer and fall of 2011, the BC Conservation Officer (CO) Service received 260 bear complaints in the Christina Lake area, in contrast to an average of 33 complaints received annually and the previous maximum number of 56 complaints from the area in 2004 [34]. Complaints in 2011 included extensive property damage, bears entering residences, and multiple vehicle break-ins. The CO Service destroyed 24 bears in 2011 in the area (not including bears killed by private landowners), as compared to the previous maximum of 4 bears killed by the CO Service in 2004 [34]. Of these, 18 were large males, which is unusual as normally 2–3 year olds and orphaned cubs or yearlings are killed as problem bears. In 2011, “Bear Aware”, a government-sponsored education program focused on managing food attractants, was offered in the community for the first time in several years [35], in an attempt to reduce conflicts in light of the feeding event.

In the second survey, over 90% of respondents were aware of bears being killed in 2011, mostly for nuisance activities and for being too close and not afraid of people. Very few participants knew how many bears had actually been killed. Most respondents based their information on local “*talk around town*” rather than personal experience or media reports. Media outlets were monitored for related stories throughout the summer, but only one report of bear deaths in late-September emerged; hence, media did not appear to influence attitudes between surveys.

One-quarter of respondents believed the bears that were killed were the food-conditioned bears. Just over half were unsure, however, and suggested that some were likely “*garbage-conditioned*” and “*dump bears*.” Several respondents pointed out that since the resident was caught feeding bears again in the summer of 2011, some bears must have been back at the property of concern. Fourteen respondents did not believe the 24 bears killed were the food-conditioned bears, including a respondent who felt very neutral towards bears in general, and suggested that “*stomach contents should have been checked*” to confirm if in fact they were the fed bears that were supposed to be targeted. 

### 3.5. Attitudes towards Pre-Hibernation Management of Bears

When asked in the first survey, before the nuisance bear activity, how supportive they were of four management options (continue feeding until hibernation, trap and relocate, trap and place in captivity, humanely kill), 62% households were somewhat or very supportive of the government decision in fall 2010 to feed until hibernation. These respondents believed it was the “*best*”, “*fairest*” and “*most viable*” option given the time of year. Further, it “*would have been cruel not to feed last fall*” and it “*saved neighbours grief*.” Several households that opposed the decision said that the feeding had gone on for a long time and that government was fully aware and did not act appropriately in the past. They suggested it was "*too late for these bears*" and that “[*they*] *are unhealthy and now the whole ecosystem is thrown off,*” as it was a “*death sentence first day they were fed.*” In contrast, a few individuals said the government should have left the people feeding the bears alone, as it had been happening without incident for years.

Many households supported the option of relocating the bears, stating that the authorities should “*give it a try*” because “*it was not the bears’ fault*”. Those opposed said relocation would not work and the bears would return or become someone else’s problem. Both captivity and humane killing (by government officials) were strongly opposed by three-quarters of households. A common view was that “*there has got to be a better option than killing*.” 

Women were more likely to support relocation than men (*p* = 0.015) and were less supportive of killing (*p* = 0.012). Hunting households were significantly less supportive of relocation in fall 2010 than non-hunting households (*p* = 0.000) and slightly less opposed to humane killing in fall 2010 than non-hunting households (*p* = 0.026), although many hunting households opposed humane killing as they felt there were better solutions. 

### 3.6. Attitudes towards Post-Hibernation Management of Bears

When asked in the first survey what the food-conditioned bears would do on emerging from hibernation, most respondents believed the bears would return to where feeding previously occurred because it had been happening for so long. Almost all respondents thought that once the bears realized no food was being provided, they would seek other human-sourced food. Some expressed concern for neighbouring properties and believed the bears would come closer to town. Although many were hopeful, few believed that bears would go back to the wild and forage naturally. Weather and food availability in spring and summer were suggested as significant factors in determining natural foraging opportunities. 

When asked their views about five possible management options for the food-conditioned bears once they emerged from hibernation, few respondents used the categories of “somewhat supportive” and “somewhat unsupportive”, so these results were combined respectively with “very supportive” and “not supportive” in Table 2. Almost two-thirds of the households said they supported prohibiting feeding and allowing the bears to live in the same area and forage naturally. Continued feeding of the bears on the property was strongly opposed. Relocation was supported by many households but a quarter of respondents were opposed to this option. Three-quarters of respondents did not support captivity, although a few felt it was preferable to killing. Humanely killing the bears in spring 2011 was not supported by more than two-thirds of respondents. A t-test for independent samples found that women were more supportive of relocation than men (*p* = 0.007), but there were no gender differences for the other options. Compared to hunting households, non-hunting households were more opposed to humane killing (*p* = 0.002) and more supportive of relocation (*p* = 0.010).

**Table 2:** Percentage of respondents in the first survey expressing different levels of support for post-hibernation management options for the food-conditioned bears ^a^.

Level of support	No feeding, natural food only	Keep feeding	Trap & Relocate	Trap & Captivity	Humanely kill
Not supportive	21%	89%	26%	75%	70%
Neutral	11%	6%	6%	6%	7%
Very supportive	65%	5%	67%	18%	23%

^a^ Results do not add to 100% in some cases because 0–3% responded with “don’t know or “no opinion”.

### 3.7. Attitudes towards Bear Management after the Summer

When contacted in the second survey, after the bear activity in summer and fall of 2011, two-thirds of respondents supported the government decision to kill the problem bears but, most acknowledged that killing was not the end they had wanted. Numerous respondents said the government should have “*keep a closer eye on the people doing it,*” since it was well known to be happening for many years. A third of respondents were not supportive of the decision to kill the bears, but these views were split between those who opposed killing and preferred “*diversionary feeding*” or believed the government “*should have relocated [the bears] immediately last year,*” and those who believed the residents “*should have been punished and bears killed right away.*” Those respondents who lived more than 10 km from the property of concern were less supportive of government actions than those who lived closer (Chi-square *p* < 0.05).

When asked what management actions they would recommend if another community faced similar challenges with intentional bear feeding, participants ranked relocation as most preferable. However, many recognized that relocation was unlikely to be successful and that bears would return or cause problems elsewhere. Allowing food-conditioned bears to continue to live in the area was ranked second, but the “*live and let live*” approach was recognized as having limits of tolerance. Killing, although disliked by many as an option, was seen as a last resort to be used when public safety becomes a concern. Diversionary feeding (intentional feeding away from original source) was generally not seen as acceptable. 

### 3.8. Respondents’ Views on Penalties for Feeding Local Bears

When asked in an open-ended question in the first survey, what an appropriate penalty would be for feeding the bears, 78% of households felt the bear-feeding residents should be held responsible somehow for their actions, although they differed on what would be appropriate. Close to half of these respondents thought a significant penalty was needed (high fine, jail time or both, seizing residents’ land), sharing this respondent’s concern: “*Yes huge fine and jail time. I love animals but I love kids too.*” But some believed fines were not realistic because the residents “*lived off the land”*. Others felt the residents should not be put in jail as the feeding was not a malicious act and because jail might not be a deterrent to future feeding. The remainder of the pro-penalty respondents said the residents should perform community service and/or pay compensation directly for future management interventions, or as one respondent explained: “*It depends on what happens to the bears.*”

However, 17% of respondents opposed any type of penalty (a few suggested a warning only), as many said they knew the residents and their good intentions. Although the feeding was seen as a misguided and reckless act, it was viewed as a non-violent crime and “*jail isn't for feeding bears*.” Some respondents felt the feeding had gone on for so long without issue; education of feeders was needed; and that although an outsider may agree with a penalty, locals may not, as one respondent expressed: “*as an insider, knowing the people, no penalty*.” Eight respondents did not want to comment on penalties as they knew the residents personally.

In the second survey, when asked about the appropriateness of the current legal charges against the feeders, attitudes toward penalty options did not change significantly (paired sample t-test *p* = 0.081), but some respondents felt there should now be some consequence, especially in light of the repeated feeding in 2011 and the high number of bears that had to be killed. 

## 4. Discussion

### 4.1. Survey Error Considerations

All surveys inherently have some degree of error. Sampling error was addressed here with a high sample size (43% response rate is high for a random digit dialing telephone survey). Some coverage error was possible, as not all homes have fixed-line telephones, although mobile telephone usage is limited in this remote geographic area. Language or hearing barriers experienced in this survey were few; the number of households that did not respond to the calls due to these constraints is unknown. Non-response error can occur when results are correlated with a type of person that is underrepresented in the sample. Gender of non-respondents and respondents was recorded and did not differ significantly. Other differences between respondents and non-respondents could not be tested. Under different circumstances, an intercept survey (initial in-person contact) would be a complementary method to gain information on non-respondents. This research used labour-intensive telephone surveys; however, face-to-face community meetings or virtual town hall meetings by telephone may be another strategy to solicit local and timely opinions.

### 4.2. Local Attitudes towards Management Options

Although this community was very experienced with bears and tolerant of their presence in general, attitudes towards bears became more negative between survey phases after increased sightings and higher problem-bear activity. These results agree with findings of other studies where increased experience of conflict can lead to more negative attitudes towards wildlife [36,37,38]. Although many respondents believed the government decision to feed the food-conditioned bears in fall 2010 was the best decision at the time, the majority opposed continued feeding in spring 2011 and wanted better government monitoring of the property in future. Consistent with studies of other species associated with human-wildlife conflict, participants who lived closest to the property with the food-conditioned bears held the most negative attitudes and were most supportive of lethal government actions after summer [39].

Relocation was the preferred outcome for most participants, although some were unsure whether it would be effective, as the bears might return, become another community’s problem, or not survive in a new environment. Captivity and diversionary feeding were not supported for this situation or future incidents of intentional bear feeding. Before the busy summer, most respondents felt that the food-conditioned bears should be killed only as a last resort if they became a threat to people. Those bears that appeared unusually determined to obtain anthropogenic foods and not leave human areas were killed by COs. Other bears that were reported as a nuisance in the local area were also trapped and killed, presumed to be the food-conditioned bears. Overall though, the community wanted another option to lethal, reactive management, especially given a lack of pro-active prevention. 

Relocation was also favoured by the public in other situations of human-bear conflict as shown by surveys of residents of Vancouver Island [40] and Colorado [9]. In the present survey, verbal comments showed that many understood the potential pitfalls of relocation, which is often be dictated by social pressure over biological merit [4]. One respondent declared that, *“generations of bears”* were *“forced into a life of crime*”, when describing the actions of the bears when they broke into homes, cabins and cars, in search of food. However, given the community’s strong desire to give these bears a “*second chance*”, an option of relocating some of the bears (juveniles only) which had less food-conditioning experience and the best possibility of success [41], could have been explored by government to increase overall support for management. Public support for relocation *versus* killing indicates a large gap between the public and experts opinion on management methods for mitigating this conflict. As relocation often ends badly for at least some animals, future public education on management strategies should better communicate the humaneness and limitations of practices in order to achieve better alignment of public and expert views [42]. 

The notion of fairness and justice were ongoing themes throughout the study, as the bears appeared to some participants as being “forced” into their problematic behaviour and thus killing them would not be fair. This demonstrates the complex motivations behind public views of wildlife management, which is generally expert-driven. There is likely no easy way to resolve this expert-public gap without a significant investment in education and attempts to create shared levels of understanding of both the public’s and expert management’s thinking on difficult conservation issues. This study can however offer a common solution, the importance of preventive action on wildlife feeding. Once wild animals have become food-conditioned, there are simply no attractive management options. Killing, even if done humanely, is seen by the public as unfair; relocation, although more palatable to the public, is seen by experts as not viable or not humane. 

A number of other potentially significant issues raised were known only by the local community. First, many respondents believed that a fear of bears was exaggerated by seasonal tourists who were less tolerant of bears and made hasty nuisance reports. Tracking resident type in nuisance bear calls could have determined whether there was a disproportionate reporting frequency reflecting a lack of experience and tolerance with bears. Further, the closure of the garbage dump was never discussed in media or in government reports, but numerous respondents wondered if any of the bears killed in 2011 were those displaced from the landfill. Marking the food-conditioned bears in fall 2010 or checking stomach contents (for the presence of dog food) of the killed bears might have provided answers to these concerns from locals.

The value of public participation in wildlife management decision-making has been well documented [7,10,43], but local attitudes may be distinct from broader public attitudes. This study suggests that local experience, including knowledge of past feeding activities and other factors such as the landfill closure, could have provided insight for management actions. If locals knew of past feeding and were on board with management strategies, they may have reported that it had restarted; officials could have then acted quicker to stop the activity before so many bears were involved, and perhaps fewer would have been killed. Separately tagging the “pot bears” and any known problem bears from the landfill would have confirmed which were targeted by the lethal actions, resolving these unanswered questions from locals and increasing support for management intervention. Future studies of contentious management dilemmas may also benefit from distinguishing local from broader societal attitudes; in this case, the Canadian public reacting online and in the media had no idea about the complexity of the feeding event or community impact. 

### 4.3. Perceptions of Wildlife Feeding and Consequences

Local residents, although clearly aware of the feeding, allowed it to continue for many years without reporting it to the authorities. Moreover, several respondents indicated, and government officials confirmed, that a number of food-conditioned bears from the same property were killed approximately 10 years earlier under the same circumstances. Further, despite the killing of at least 24 bears, attitudes towards penalties shifted little as the need for consequences was emphasized between the two surveys. It is notable that many participants did not regard the illegal feeding of bears as a serious offence that merited significant penalties; they believed the individuals feeding the bears were misguided but well-motivated and generous, rather than as engaging in behaviour that harms animals. Hence, participants did not favour serious punishment of the offenders and eventually the justice system concurred [44]. In fact, the feeding became a focus for enforcement only when the perpetrators were investigated for an entirely different offense. 

This case highlights a significant problem with the public view of wildlife feeding. As seen here, feeding inevitably leads to poor outcomes for the animals—death, relocation, or captivity—but the general “*laissez-faire*” attitude towards feeding all wildlife demonstrates that the public does not seem to see it on par with other forms of animal harm [48]. Thus, there is a major need for education so that people understand that illegal feeding of such wildlife is a serious form of harm to animals. The only approach is for both the authorities and the public to not tolerate such wildlife feeding and exercise the same vigilance that would be directed to other forms of animal harm (e.g., neglect, abuse). Moreover, although much research has focused on measuring the success of managing problem bear behaviour [3,49], more research is needed on measuring the success of deterrents for human behaviour, like public education [50] and attitudinal changes [51], as well as the effectiveness of enforcement and penalties [52].

This case highlights a significant problem with the public view of wildlife feeding. As seen here, feeding inevitably leads to poor outcomes for the animals—death, relocation, or captivity—but the general “*laissez-faire*” attitude towards feeding all wildlife demonstrates that the public does not seem to see it on par with other forms of animal harm [48]. Thus, there is a major need for education so that people understand that illegal feeding of such wildlife is a serious form of harm to animals. The only approach is for both the authorities and the public to not tolerate such wildlife feeding and exercise the same vigilance that would be directed to other forms of animal harm (e.g., neglect, abuse). Moreover, although much research has focused on measuring the success of managing problem bear behaviour [3,49], more research is needed on measuring the success of deterrents for human behaviour, like public education [50] and attitudinal changes [51], as well as the effectiveness of enforcement and penalties [52].

## 5. Conclusion

In contrast to wildlife experts, most participants supported relocating the food-conditioned bears or allowing them to remain in the area ahead of killing, despite the acknowledged problems of relocation. The general finding that people prefer non-lethal methods to lethal alternatives in contrast to experts is not surprising and adds to growing literature that highlights the disconnect between the public and experts on wildlife management issues. The strongly polarised management options of relocation and killing signal a need to address the current limitations of mitigating conflict with bears, and explore the use of more deterrents (for both people and bears) to expand the overall management toolbox. Intentional wildlife feeding should be one management issue where the public and experts agree on the importance of preventive and unified action. Yet, in this case most locals were aware of the years of feeding but did not report it, evidently failing to see it as a serious form of harm, even after many bears had been killed. Contrary to the “save the bears” websites and social media campaigns which mostly reflected idealism, local attitudes were diverse, more contextualized and pragmatic, as residents seemed more aware of the consequences. In fact, locals were not merely pragmatic, seeking the best outcomes for themselves; they also included notions of fairness and justice for the bears, noting that it was not the bears’ fault. Given that managers want to retain community support and ensure reporting of illegal feeding, lethal solutions should be a last resort and proactive alternatives (*i.e.*, evaluative education and strict enforcement of feeding penalties) should be standard tools for human-wildlife conflicts. 
